# Spotted Fever Group *Rickettsia* Infecting Ticks (Acari: Ixodidae), Yak (*Bos grunniens*), and Tibetan Sheep (*Ovis aries*) in the Qinghai–Tibetan Plateau Area, China

**DOI:** 10.3389/fvets.2021.779387

**Published:** 2022-02-08

**Authors:** Yong-Cai He, Ji-Xu Li, Ya-Li Sun, Ming Kang, Hong-Xuan He, Yun-Hai Guo, Ping Ma, Yao-Ping Wei, Rui-Shan Li, Wang-Kai Chen, Zhi-Hong Chen, Jing Li, Tong-Sheng Qi, Jin-Fang Yang, Qing-Xun Zhang, Ye Wang, Jin-Shan Cai, Quan-Bang Zhao, Guang-Wei Hu, Ji-Yong Chen, Ying Li

**Affiliations:** ^1^State Key Laboratory of Plateau Ecology and Agriculture, Qinghai University, Xining, China; ^2^College of Agriculture and Animal Husbandry, Qinghai University, Xining, China; ^3^National Research Center for Wildlife-Born Diseases, University of Chinese Academy of Sciences, Beijing, China; ^4^National Institute of Parasitic Diseases Chinese Center for Disease Control and Prevention, Shanghai, China; ^5^Qinghai Provincial Center for Animal Disease Control and Prevention, Xining, China; ^6^Animal Disease Prevention and Control Center of Yushu, Yushu, China

**Keywords:** Spotted Fever Group *Rickettsia*, *Haemaphysalis qinghaiensis*, yak, Tibetan sheep, Qinghai-Tibetan Plateau Area

## Abstract

The Qinghai–Tibet Plateau Area (QTPA) has a complex natural ecosystem, causing a greatly increased risk of spreading various tick-borne diseases including rickettsial infections, which are regarded as one of the oldest known vector-borne zoonoses. However, the information of one of its pathogen, spotted fever group *Rickettsia* (SFG *Rickettsia*), is limited in tick vectors and animals in this area. Therefore, this study focused on the investigation of SFG *Rickettsia* in tick vectors, yaks (*Bos grunniens*), and Tibetan sheep (*Ovis aries*) in the QTPA. A total of 1,000 samples were collected from nine sampling sites, including 425 of yaks, 309 of Tibetan sheep, 266 of ticks. By morphological examination, PCR, and sequencing, we confirmed the species of all collected ticks. All tick samples, all yak and Tibetan sheep blood samples were detected based on SFG *Rickettsia ompA* and *sca4* gene. The results showed that all tick samples were identified to be *Haemaphysalis qinghaiensis*, and the positive rates of SFG *Rickettsia* were 5.9% (25/425), 0.3% (1/309), and 54.1% (144/266) in yaks, Tibetan sheep, and ticks, respectively. All positive samples were sequenced, and BLASTn analysis of the *ompA* gene sequences of SFG *Rickettsia* showed that all positive samples from animals and ticks had 99.04–100% identity with yak and horse isolates from Qinghai Province, China. BLASTn analysis of the *sca4* gene sequences of SFG *Rickettsia* showed that all positive samples had 97.60–98.72% identity with tick isolates from Ukraine. In addition, the phylogenetic analysis showed that all the SFG *Rickettsia ompA* and *sca4* sequences obtained from this study belong to the same clade as *Rickettsia raoultii* isolated from livestock and ticks from China and other countries. Molecularly, this study detected and characterized SFG *Rickettsia* both in the tick vectors and animals, suggesting that the relationship between SFG *Rickettsia*, tick species and animal hosts should be explored to understand their interrelationships, which provide a theoretical basis for preventing control of this pathogen.

## Introduction

*Rickettsia* spp. (Rickettsiales: Rickettsiaceae) are obligate intracellular bacteria that causes rickettsioses, which is one of the oldest known vector-borne zoonoses diseases (1). The genus *Rickettsia* consists of four members: spotted fever group (SFG) rickettsiae, typhus group rickettsiae, the *Rickettsia bellii* group, and the *Rickettsia* canadensis group (2). At present, 26 SFG *Rickettsia* species had been reported, 16 of which were associated with human diseases (3, 4). In addition to the typical triad of fever, rash, and headache, the classic symptoms of new cases of *Rickettsia* varied by type, and in some cases, the typical symptoms might not appear or be ignored (4). Therefore, the clinical diagnosis of rickettsioses is a huge challenge for both physicians and veterinarians (3, 4).

SFG *Rickettsia* includes more than 20 different species, including pathogenic species that cause human and animal incidence, and nonpathogenic species (5). In Asia, a variety of SFG *Rickettsia* have been reported, and these rickettsiae are mostly distributed in border areas of various countries. In the Russian Far East, Kazakhstan, northern China, and Mongolia, *R. sibirica* and *R. raoultii* are widely distributed (4). In China, 11 SFG *Rickettsia* species have been detected in tick vectors and animals, some of which have been characterized as human pathogens (6). In previous reports, SFG *Rickettsia* was detected in a variety of ticks, including *Haemaphysalis qinghaiensis* in Yunnan Province and Harbin Province in China (5, 7, 8). However, there are few reports of the determination of SFG *Rickettsia* in *H. qinghaiensis* in the Qinghai–Tibetan Plateau Area (QTPA) (7, 9).

The QTPA is well known for the largest and highest plateau in the world, which is located in northwestern China and with a unique natural ecosystem (7). Yaks and Tibetan sheep are indigenous animals on the QTPA, which are the important sources of milk, meat, fur, skins, and dung in the area, contributing to the local economic development and the lives of herdsmen (10). Although SFG rickettsioses in livestock have been reported in this area, the information on these pathogens are limited (6, 11). *H. qinghaiensis* is a common and endemic three-host tick on the QTPA, which is easy to parasitize on Tibetan sheep, yaks, sheep, goats, and other domestic animals to transmit a variety of diseases (9). Therefore, the existence of *H. qinghaiensis* is a potential threat to the animal husbandry in the QTPA (12).

This study aimed to determine the existence and molecular characteristics of SFG *Rickettsia* from yaks, Tibetan sheep, and tick vectors in the QTPA, so as to further understand the prevalence of SFG *Rickettsia* and clarify the interaction between ticks and animals.

## Materials and Methods

### Sample Collection and DNA Extraction

**Figure 1:**
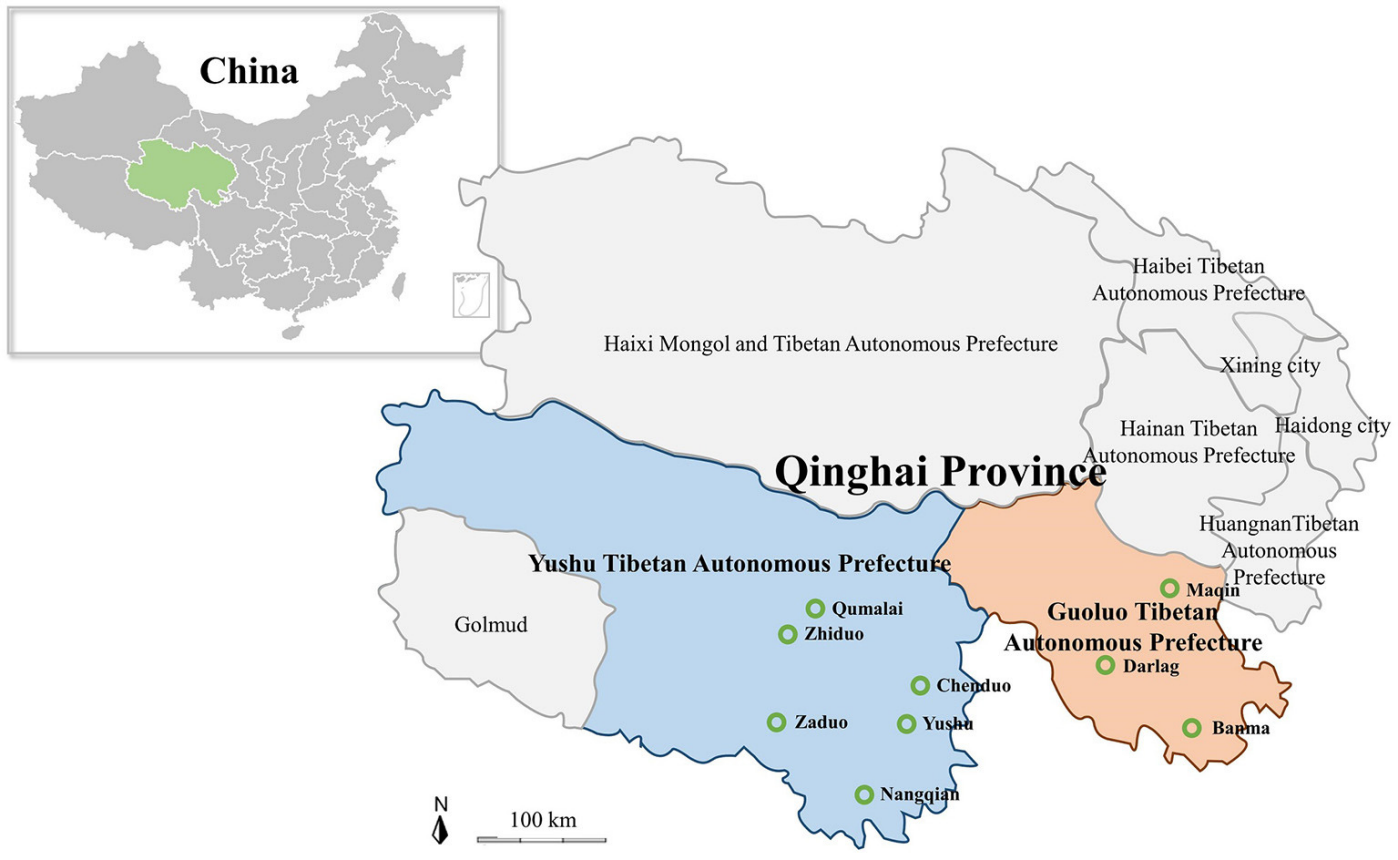
Map of the Qinghai–Tibetan Plateau Area and Qinghai Province showing the sampling sites. The green hollow circle indicates the location of the sample collection in this study.

In the present study, all of 1,000 samples including yak and Tibetan sheep blood samples, and tick samples were collected from nine cites of Yushu Tibetan Autonomous Prefecture (Yushu) and Guoluo Tibetan Autonomous Prefecture (Guoluo) in Qinghai Province of QTPA (Figure 1). The 734 blood samples of yaks and Tibetan sheep were collected in tubes containing an EDTA anticoagulant, and genomic DNA was extracted using the MagPure Blood DNA KF Kit (Magen, China) according to the manual of the manufacturer. The 266 tick samples were collected, some of which were engorged ticks collected on the surface of yaks and Tibetan sheep, and the rest of ticks were collected by dragging the grasslands with a flannel cloth. DNA was extracted by using the MagPure Mollusc DNA KF Kit (Magen, China) and stored at −80°C until further use.

### Identification of Ticks and Detection of Pathogens

Ticks were identified by the mitochondrial 16S rRNA gene (forward primer 5′-CTG CTC AAT GAT TTT TTA AAT TGC TGT GG-3′, reverse primer 5′-CCG GTC TGA ACT CAG ATC AAG T-3′) and morphological characteristics (13, 14). Outer membrane protein A (*ompA*) gene and surface cell antigen 4 (*sca4*) gene were used to investigate the molecular characterization of the SFG *Rickettsia* by using genus-specific primers (forward primer 5′-GCT TTA TTC ACC ACC TCA AC-3′ and reverse primer 5′-TRA TCA CCA CCG TAA GTA AAT-3′; D767f: 5′-CGA TGG TAG CAT TAA AAG CT-3′ and D1390r: 5′-CTT GCT TTT CAG CAA TAT CAC-3′) (15, 16). PCR reaction volume of 10 μl containing 3 μl of DNA template, 0.5 μl each of forward and reverse primer (100 μM), 0.1 μl of Taq polymerase (0.5 U; New England BioLab, USA), 0.2 μl of deoxyribonucleotide triphosphate (200 μM; New England BioLab, USA), 1 μl of 10× ThermoPol Reaction Buffer (New England BioLab, USA), and double-distilled water to a volume of 10 μl. Positive yak DNA samples of *Rickettsia* spp. *ompA* gene from our previous study were used as positive controls, and double-distilled water was used as a negative control (11).

### Sequencing and Phylogenetic Analyses

All PCR positive tick and pathogen samples were purified using EasyPure® Quick Gel Extraction Kit (TransGen, China) and cloned into *E. coli* DH5α using the PMD^TM^ 19-T Vector Cloning Kit (TaKaRa, Japan). At least two positive clones from each positive sample were selected for sequencing in Sangon Biotech (Shanghai) Co., Ltd. Nucleotide sequence identities were determined by performing GenBank BLASTn analysis (https://blast.ncbi.nlm.nih.gov/Blast.cgi). Phylogenetic trees based on the obtained sequences were constructed using the neighbor-joining method, and genetic distance matrix among various *Rickettsia* clades in a phylogenetic tree were calculated using the maximum composite likelihood model in MEGA7.0 (17).

### Statistical Analysis

The Chi-square test was used to evaluate the difference in prevalence between different parameters. Exposure variables included prefecture (Guoluo and Yushu), altitude (3,000–4,000 and 4,000–5,000 m), and animals (yak and Tibetan sheep). When the *p*-values were lower than 0.05, the result was considered to be of statistically significant difference (https://www.mathsisfun.com/data/chi-square-calculator.html).

## Results

### Identification of Ticks

**Figure 2:**
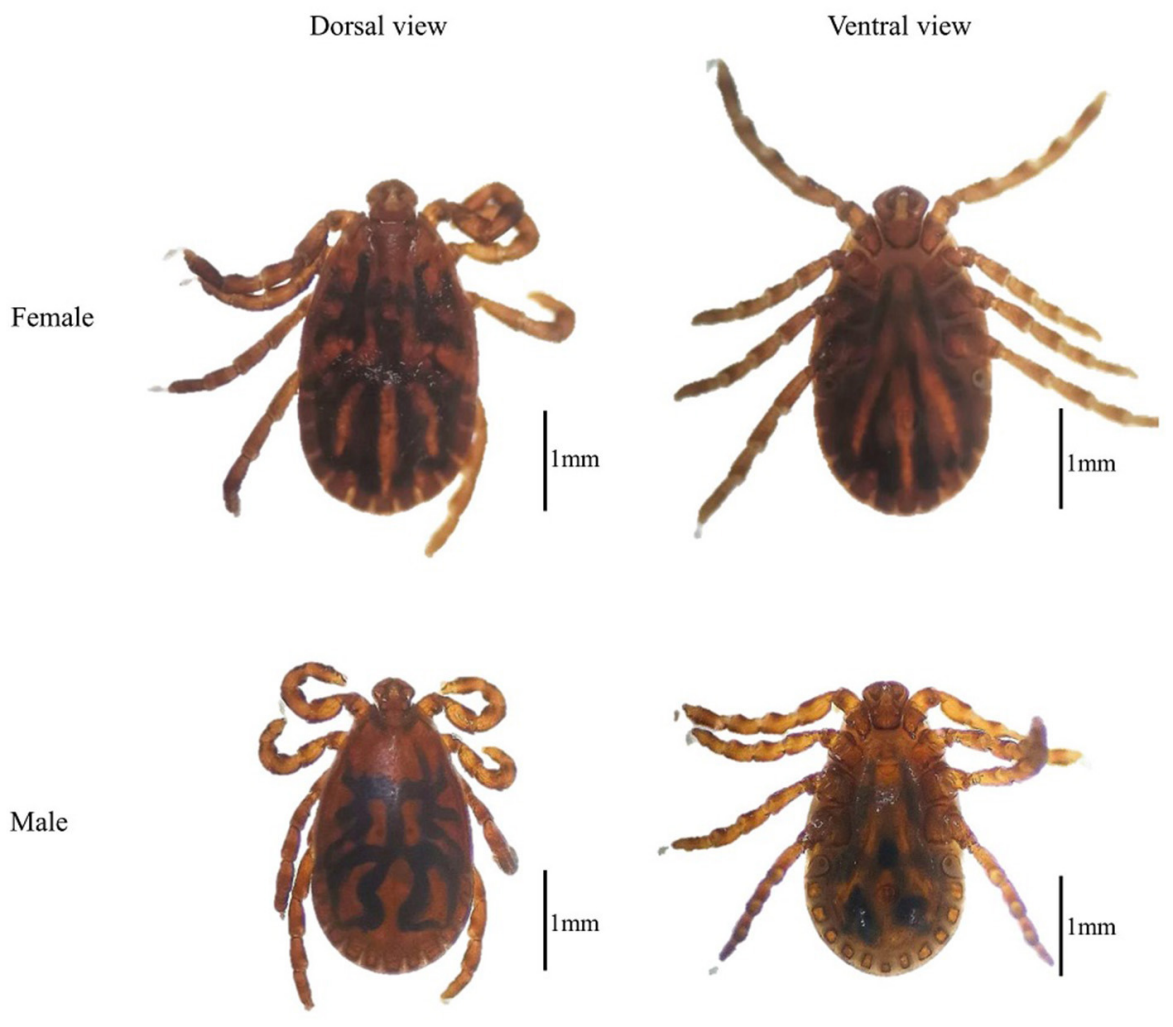
The dorsal view and ventral view of *Haemaphysalis qinghaiensis*.

**Figure 3:**
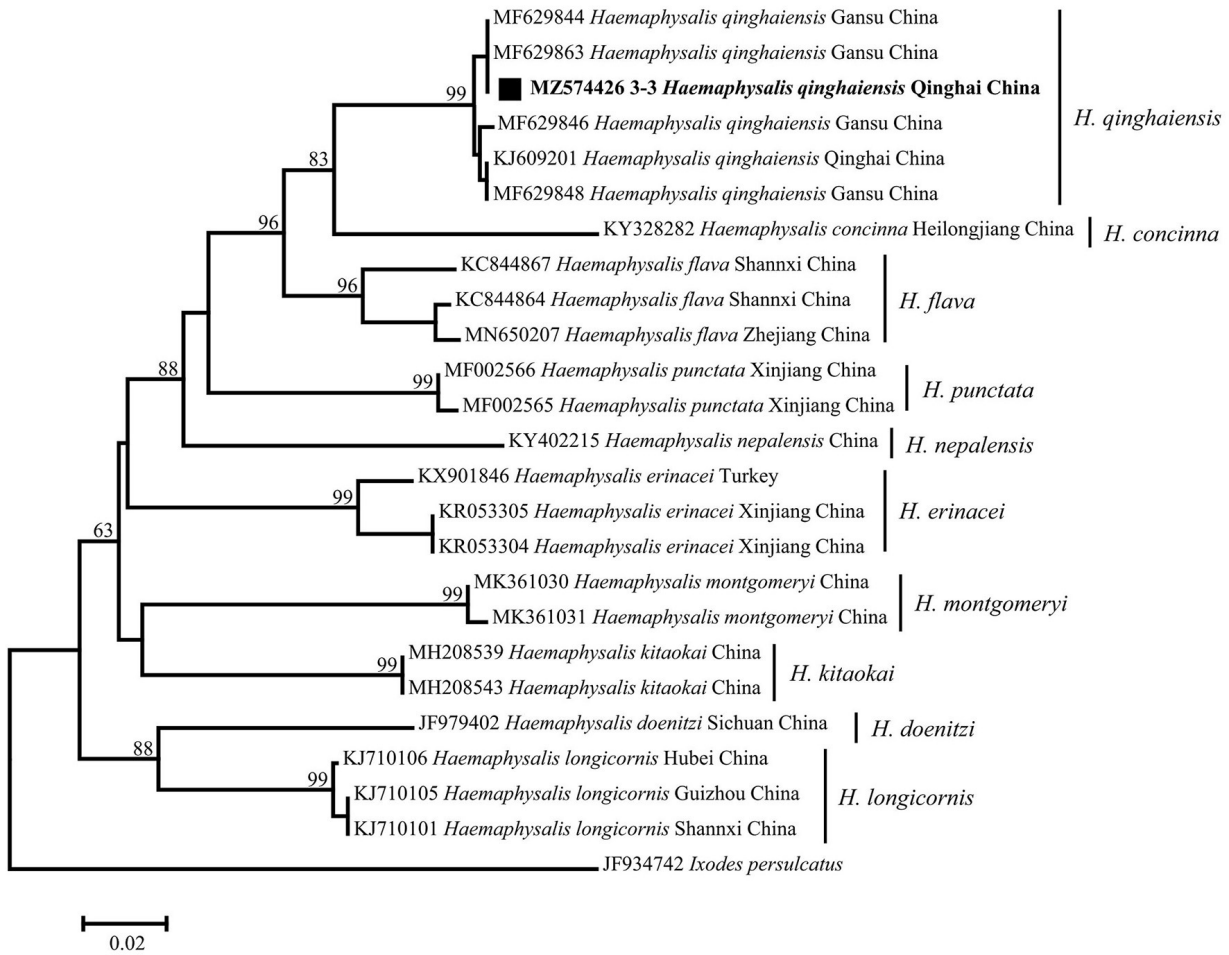
Phylogenetic tree based on the mitochondrial 16S rRNA gene (460 bp) of *Haemaphysalis* spp. obtained in this study. Bootstraps analysis was performed with 500 replicates. The solid square indicates sequence from this study. One sequence from this study is bolded. *Ixodes persulcatus* (JF934742) was used as outgroup.

By morphological examination, all the tick samples were identified as *Haemaphysalis* (Figure 2) (14); then the mitochondrial 16S rRNA gene target was used as an identification molecular marker, and 30 tick specimens were randomly selected for sequencing. By BLASTn analysis and phylogenetic analysis, the evidence demonstrated that there is an extensive homology between the *Haemaphysalis* species 16S rRNA gene sequence and the published sequence of *H. qinghaiensis* (MF629844) from Gansu Province, China, and grouped with the *H. qinghaiensis* clades (Figure 3 and Table 1).

**Table 1:** DNA sequences of spotted fever group (SFG) *Rickettsia* obtained in this study.

**Obtained sequences**					**The closest BLASTn match**		
**Tick and tick borne pathogens**	**Sample**	**Target gene**	**Accession number**	**Length (bp)**	**Identity (%)**	**Species**	**Accession number (host, country)**
*Haemaphysalis* spp.	tick	16S rRNA	MZ574426	454	100	*H. qinghaiensis*	MF629844 China
Rickettsia spp.	Tick	*ompA*	MZ130274	209	100	*R. raoultii*	MN394801 yak China
	Tick		MZ130275	207	100	*R. raoultii*	MN394801 yak China
	Tick		MZ130276	209	100	*R. raoultii*	MN394800 yak China
	Tick		MZ130277	209	99.52	*R. raoultii*	MN394801 yak China
	Tick		MZ130278	209	99.04	*R. raoultii*	MN394801 yak China
	Tick		MZ130279	209	99.52	*R. raoultii*	MN394801 yak China
	Tick		MZ130280	209	99.52	*R. raoultii*	MN394801 yak China
	Tick		MZ130281	209	99.52	*R. raoultii*	MN394801 yak China
	Tick		MZ130282	208	99.04	*R. raoultii*	MN394800 yak China
	Tick		MZ130283	209	100	*R. raoultii*	MN394800 yak China
	Tick		MZ130284	209	100	*R. raoultii*	MN394800 yak China
	Tick		MZ130285	209	100	*R. raoultii*	MN394801 yak China
	Yak		MZ130267	209	100	*R. raoultii*	MK307883 horse China
	Yak		MZ130268	209	100	*R. raoultii*	MN394797 yak China
	Yak		MZ130269	209	100	*R. raoultii*	MN394797 yak China
	Yak		MZ130270	209	100	*R. raoultii*	MK307883 horse China
	Yak		MZ130271	209	99.52	*R. raoultii*	MN394798 yak China
	Yak		MZ130272	209	99.52	*R. raoultii*	MN394797 yak China
	Tibetan sheep		MZ130273	209	100	*R. raoultii*	MK307883 horse China
	Tick	*sca4*	OL621221	624	98.72	*R. raoultii*	KP768191 tick Ukraine
	Tick		OL621222	624	98.56	*R. raoultii*	KP768191 tick Ukraine
	Tick		OL621223	624	98.40	*R. raoultii*	KP768191 tick Ukraine
	Tick		OL621224	624	97.60	*R. raoultii*	KP768191 tick Ukraine
	Tick		OL621225	624	97.92	*R. raoultii*	KP768191 tick Ukraine

### Infection Rates of Spotted Fever Group *Rickettsia*

In the current study, the overall infection rate of SFG *Rickettsia* was 17% (170/1,000), including the infection rate of 51.35% (76/148) in animal-derived engorged ticks, 57.6% (68/118) in free ticks, 5.9% (25/425) in yaks, and 0.3% (1/309) in Tibetan sheep (Table 2).

**Table 2:** The positive samples of spotted fever group (SFG) *Rickettsia* in ticks and animals in this study.

**Prefectures**	**Sampling sites**	**Altitude (m)**	**Number of positive samples (Infection rate %)**				
			Tick-E^*^	Tick-F^*^	**Yak**	**Tibetan sheep**	**Total**
Yushu	Yushu	4,117–4,317	6/15 (40.0)	20/38 (52.6)	0/53	0	26/106 (24.5)
	Zhiduo	4,171.4	17/37 (46.0)	0	0/30	0	17/67 (25.4)
	Zaduo	4,290	0	0	0/33	0	0/33
	Chenduo	4,000	0	10/17 (58.8)	0/29	0/25	10/71 (14.1)
	Nangqian	3,640–3,920	0	13/18 (72.2)	0/48	0/51	13/117 (11.1)
	Qumalai	4,279	0	0	3/22 (13.6)	1/50 (2.0)	4/72 (1.4)
	Total		23/52 (44.2)	43/73 (58.9)	3/215 (1.4)	1/126 (0.8)	70/466 (15.0)
Guoluo	Banma	3,623–3,877	0	15/25 (60.0)	10/78 (12.8)	0	25/103 (24.3)
	Darlag	4,130	2/9 (22.2)	0	0/35	0/51	2/95 (2.1)
	Maqin	3,859	51/87 (58.6)	10/20 (50.0)	12/97 (12.4)	0/132	73/336 (21.7)
	Total		53/96 (55.2)	25/45 (55.6)	22/210 (10.5)	0/183	100/534 (18.7)
Total			76/148 (51.35)	68/118 (57.6)	25/425 (5.9)	1/309 (0.3)	170/1,000 (17.0)

* *E, engorged ticks collected from yaks or Tibetan sheep in this study; F, free ticks on the grass*.

Statistical analysis showed that for yaks, the infection rate of SFG *Rickettsia* in Guoluo was significantly higher than that of Yushu (*p* = 0.0002), and the infection rate at altitudes of 3,000–4,000 m was significantly higher than that at altitudes of 4,000–5,000 m (*p* = 0.0005) (Table 3). Moreover, altitude also was a significant impact factor on the total infection rate, such as the infection rate of SFG *Rickettsia* at an altitude of 3,000–4,000 m was remarkably higher than that of 4,000–5,000 m (*p* = 0.0183) (Table 3). However, there was no significant difference in the infection rate of SFG *Rickettsia* from engorged *H. qinghaiensis* divided from yaks and Tibetan sheep, and also no significant difference in the infection rate of SFG *Rickettsia* of free *H. qinghaiensis* and engorged those in this study (*p* > 0.05) (Table 4).

**Table 3:** The patterns of infection rates of spotted fever group (SFG) *Rickettsia* in ticks, yaks, and Tibetan sheep grouped by prefecture and altitude of the sampling sites.

**Parameter**	**Number of positive samples (infection rate %)**				
	Tick-E^*^	Tick-F^*^	**Yak**	**Tibetan sheep**	**Total**
**Prefecture**					
Yushu	23/52 (44.2)	43/73 (58.9)	3/215 (1.4)	1/126 (0.8)	70/466 (15.0)
Guoluo	53/96 (55.2)	25/45 (55.6)	22/210 (10.5)	0/183 (0)	100/534 (18.7)
*p*-Value	0.4644	0.8526	0.0002	0.2292	0.1895
**Altitude (m)**					
3,000–4,000	51/87 (58.6)	38/63 (60.3)	22/223 (9.9)	0/183 (0)	111/556 (20.0)
4,000–5,000	25/61 (41.0)	30/55 (54.5)	3/202 (1.5)	1/126 (0.8)	59/444 (13.3)
*p*-value	0.2253	0.7424	0.0005	0.2292	0.0183

* *E, engorged-ticks collected from yaks or Tibetan sheep in this study; F, free ticks on the grass*.

**Table 4:** The infection rate of spotted fever group (SFG) *Rickettsia* in engorged ticks.

**Prefectures**	**Number of positive engorged tick samples (infection rate %)**		
	**Yak**	**Tibetan sheep**	***p*-Value**
Yushu	17/37 (46.0)	6/15 (40.0)	0.8061
Guoluo	3/8 (37.5)	50/88 (56.8)	0.5503
Total	20/45 (44.4)	56/103 (54.4)	0.5231

### Sequencing and Phylogenetic Analysis of Spotted Fever Group *Rickettsia ompA* Gene

**Figure 4:**
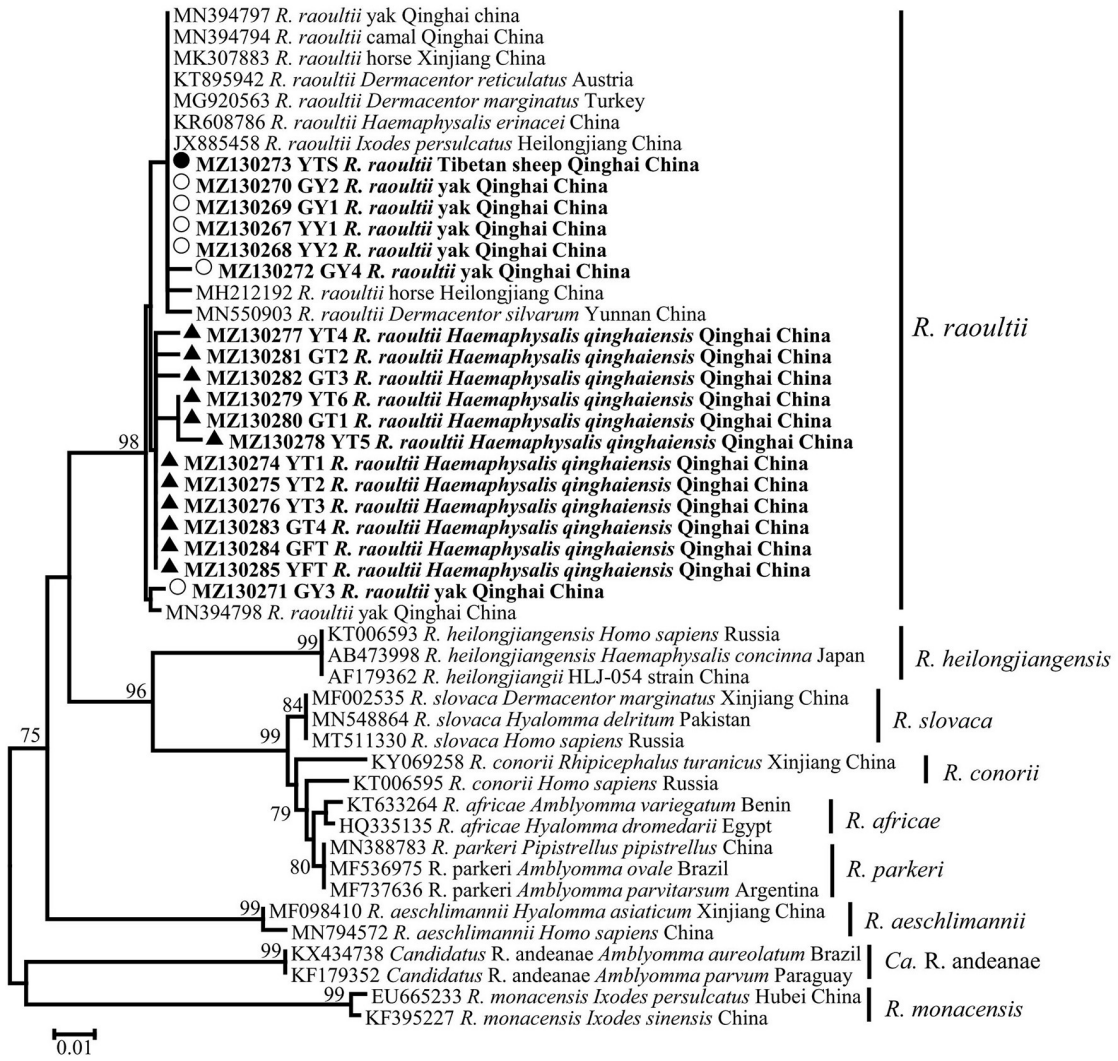
Phylogenetic tree based on *ompA* particle sequences (209/212 bp) of spotted fever group (SFG) *Rickettisa* obtained in this study. Bootstraps analysis was performed with 500 replicates. The solid circle indicates sequences from Tibetan sheep, the empty circle indicates sequences from yaks, and the solid triangle indicate sequences from *Haemaphysalis qinghaiensis*. All sequences from this study are bolded.

In this study, a total of 19 SFG *Rickettsia ompA* gene sequences (MZ130267–MZ130285) were obtained, and the identity between each sequence was 94.2−100%, and the identity was 99.04–100% between MK307883, MN394797, MN394798, MN394801, and MN394800. The identity was 95.7–98.6% between the sequences of ticks and those of animals. Phylogenetic analysis of SFG *Rickettsia ompA* gene revealed that 19 sequences were in the same clade as *R. raoultii* isolated from horse, camel, *Haemaphysalis erinacei, Ixodes persulclcatus*, and three *Dermacentor* species from China, Austria, and Turkey (Figure 4). Genetic distance among various *Rickettsia* clades was 1–21% and *R. raoultii* with 8–13% genetic distance with other rickettsiae (Table 5).

**Table 5:** Genetic distance matrix among various *Rickettsia* species in a phylogenetic tree based on the *ompA* gene in this study.

**Rickettsial species**	**Evolutionary divergence over sequence pairs between rickettsial species (%)**								
	**1**.	**2**.	**3**.	**4**.	**5**.	**6**.	**7**.	**8**.	**9**.
1. *R. raoultii*									
2. *R. slovaca*	8								
3. *R. aeschlimannii*	9	13							
4. *R. parkeri*	9	1	14						
5. *R. conorii*	10	1	14	1					
6. *Candidatus* R. andeanae	12	14	16	14	15				
7. *R. africae*	9	1	14	1	2	15			
8. *R. heilongjiangensis*	9	8	14	8	10	15	9		
9. *R. monacensis*	13	19	15	20	20	16	21	18	

### Sequencing and Phylogenetic Analysis of Spotted Fever Group *Rickettsia sca4* Gene

**Figure 5:**
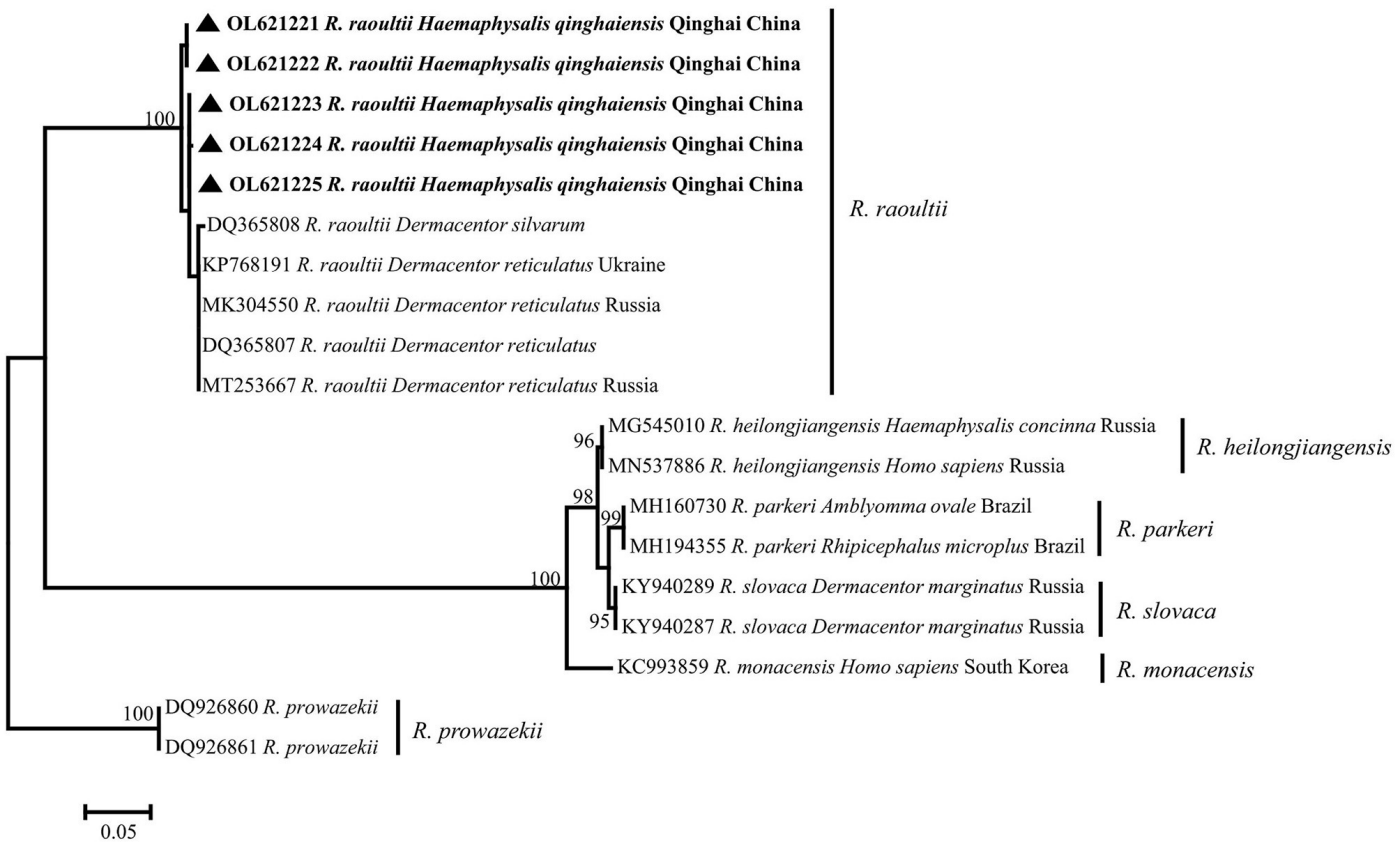
Phylogenetic tree based on *sca4* particle sequences (624 bp) of spotted fever group (SFG) *Rickettsia* obtained in this study. Bootstraps analysis was performed with 500 replicates. The solid triangle indicates sequences from *Haemaphysalis qinghaiensis*. All sequences from this study are bolded.

All of five SFG *Rickettsia sca4* gene sequences (OL621221–OL621225) were obtained, and the identity between each sequences was 98.6−99.8%, and the identity was 97.60−98.72% in KP768191. Phylogenetic analysis of SFG *Rickettsia sca4* gene revealed that five sequences were in the same clade as *R. raoultii* isolated from *Dermacentor reticulatus* from Ukraine (Figure 5). Genetic distance among various *Rickettsia* clades was 1−42%, *R. raoultii* with 2−25% genetic distance with other rickettsiae (Table 6).

**Table 6:** Genetic distance matrix among various *Rickettsia* species in a phylogenetic tree based on *sca4* gene in this study.

**Rickettsial species**	**Evolutionary divergence over sequence pairs between rickettsial species (%)**					
	**1**.	**2**.	**3**.	**4**.	**5**.	**6**.
1. *R. raoultii*						
2. *R. heilongjiangensis*	2					
3. *R. parkeri*	3	3				
4. *R. monacensis*	24	10	6			
5. *R. slovaca*	2	1	1	5		
6. *R. prowazekii*	25	28	26	42	23	

## Discussion

*Haemaphysalis qinghaiensis* was first discovered in Huangyuan County, Qinghai Province in 1980, and was subsequently discovered in Gansu, Sichuan, Ningxia, Yunnan, Tibet provinces of China (14, 18). There was no record abroad. In nature, a complete life history of *H. qinghaiensis* is generally 3 years (14). It was the dominant tick species in Qinghai Province of the QTPA, China, and it is mainly parasitic on various domestic animals, and as the vector of *Theileria* sp. and *Babesia* sp. (12, 14). Therefore, *H. qinghaiensis* and its transmitted diseases were the main economic burden of animal husbandry in Qinghai Province in the QTPA (12).

In the past 30 years, the scope and importance of pathogenic rickettsiae associated with ticks had increased dramatically (19). Most of the pathogenic *Rickettsia* species discovered had been found in ticks, and ticks were considered to be the most important vector and reservoir host of *Rickettsia* in almost every region of the word (4, 20, 21). Later, they were discovered to be related to disease symptoms in humans, such as headache, arthralgias, multiple eschars, and meningoencephalitis (22, 23). Although ticks were parasitic on humans appearing to be opportunistic, the risk of *Rickettsia* infection was greatly increased among herders and livestock living on the Tibetan Plateau, where ticks are widely distributed.

*R. raoultii* is a bacterium that was first isolated in 1999 from *D. nuttalli* and *Rhipicephalus pumilio* and was described as a novel species in 2008 (24, 25). In China, the first reported case of *R. raoultii* in 2012 was from *D. silvarum* in Xinjiang Province (26). In a previous report of Wen et al., *R. raoultii* was detected in at least 13 species of ticks belonging to six genera, including *Dermacentor, Ixodes, Rhipicephalus, Haemaphysalis, Amblyomma*, and *Hyalomma* (27). Moreover, *R. raoultii* also was detected in animals, such as yaks, Bactrian camel (*Camelus bactrianus*), and red fox (*Vulpes vulpes*) (11, 28). Importantly, *R. raoultii* was considered to be a human pathogen that had been shown to cause Tick-borne lymphadenopathy (TIBOLA) and *Dermacentor*-borne necrosis erythema and lymphadenopathy (DEBONEL) (29), and it should be emphasized that the cases had also been reported in China (30, 31).

*ompA* is considered to be a good candidate gene for phylogenetic analysis of most SFG representatives, with a higher degree of interspecies variation than 16S rRNA and citrate synthase-encoding gene (*gltA*), and *ompA* is present in almost all SFG members (32). *sca4* also has been used to rapidly and reliably differentiate members of the genus *Rickettsia* (16). Thus, *ompA* and *sca4* gene fragments were used to specifically detect rickettsial infection in ticks in many studies, such as Ishikura et al. (33), Orkun et al. (34), Taylor et al. (35), Jiang et al. (36), Kooshki et al. (37) and Nadim et al. (38). Therefore, in the current study, *ompA* and *sca4* were selected as the target genes to detect SFG *Rickettsia* in yak, Tibetan sheep blood samples, and tick samples. This study results showed that SFG *Rickettsia* infection was detected in both tick and animal blood samples. By sequencing and BLASTn analysis, the evidence demonstrated that there were 99.04–100% sequence identities between obtained SFG *Rickettsia ompA* sequences (MZ130267–MZ130285) and *R. raoultii* (MK307883, MN394797, MN394798, MN394801, and MN394800) isolated from China. Meanwhile, BLASTn analysis of *sca4* sequences showed 97.60–98.72% similarity with *R. raoultii* (KP768191) isolated from *D. reticulatus* from Ukraine. Furthermore, phylogenetic analysis showed that the current study obtained DNA sequences that formed an independent clade with *R. raoultii* from China, Austria, Turkey, Ukraine, and Russia, with a high bootstrap value. The genetic distance matrix based on *Rickettsia ompA* and *sca4* genes showed that the genetic distances between *R. raoultii* and other rickettsiae were 8–13% and 2–25%, respectively (Tables 5, 6). In previous studies, this pathogen was also reported in various provinces such as Jilin (39), Heilongjiang (30), Gansu (40), Shaanxi (40), Xinjiang (26), and Yunnan (5), which suggests that the geographic distribution of *R. raoultii* covered most of China.

Molecular biology detection revealed that bacteria of genus *Rickettsia* were distributed all over the world, which was related to the geographical distribution of their vectors (4). Ticks, as the most important vectors of rickettsiae, were distributed throughout the world; the risk of rickettsioses in a given region might depend on the presence and biotopes of specific species of ticks (41). Among Asian countries, *R. sibirica* was endemic in most of the areas of Russia, China, Mongolia, and Kazakhstan, and was mainly transmitted by the *Dermacentor* tick (3). In addition, it was reported in Russia and China that *R. heilongjiangensis* isolated from *H. concina* and *D. silvarum*, and the cases caused by this *Rickettsia* species, had also been reported in Japan (1, 42). *R. japonica* was also found in Japan and was transmitted by a variety of *Haemaphysalis* ticks, other *Ixodes* and *Dermacentor* ticks (1). *Rickettsia* strains closely related to *R. japonica* were detected in ticks in South Korea and northern Thailand (43, 44). In Turkey, researchers detected different SFG *Rickettsia* in different ticks; *R. aeschlimanii* was detected in *H. marginatum, R. raoultii* and *R. slovaca* were detected in *D. marginatus*, and *R. hoogstraalii* in *H. parva* (45). In India, *R. conorii* subsp. *indica* was widespread, and the cases were also reported in Laos and Sri Lanka (1). Ticks are the most important external parasites of livestock and wildlife in Iran. A total of 46 species had been reported (46), in which *Argas persicus* was the important ectoparasite of poultry in Iran. In previous studies, *R. Hoogstraalii* (37) and SFG *Rickettsia* (47) had been detected in *Argas persicus*. In addition, the epidemiological investigation on SFG *Rickettsia* of *D. marginatus* Sulzer, *H. sulcata, H. inermis*, and *Hyalomma asiaticum* in Iran was reported (38, 47).

In the current study, the infection rates of SFG *Rickettsia* in free *H. qinghaiensis*, engorged *H. qinghaiensis*, yaks, and Tibetan sheep were 57.6%, 51.35%, 5.9%, and 0.3%, respectively. Studies had reported the detection of *R. raoultii* in yaks, camels, and several ticks in Qinghai Province (7, 11). Combined with the experimental results of this study, it was suggested that *H. qinghaiensis* might be the host of this pathogen, but because the infection rate of this pathogen between animals and ticks was remarkably different, therefore, whether the pathogen could be transmitted by *H. qinghaiensis* needed further study. Meanwhile, a phylogenetic tree showed that the *ompA* sequences of SFG *Rickettsia* isolated from *H. qinghaiensis* in Qinghai belonged to the same clade as the reported one, while the *ompA* gene sequences of SFG *Rickettsia* isolated from animals (except MZ130271) were in same clade as those of other tick species (including *Dermacentor* and *Ixodes*). In addition, Han et al. also detected this pathogen in several *Dermacentor ticks* in Qinghai Province (7). According to a previous report (48) that *R. raoultii* was closely associated with *Dermacentor* spp., we suspected that this pathogen isolated from animals in this study was not mainly transmitted by *H. qinghaiensis*. The high infection rate of this pathogen in *H. qinghaiensis* suggested that *H. qinghaiensis* might be the reservoir of *R. raoultii*.

In the current study, the survey results showed that the infection rate of SFG *Rickettsia* in yaks was affected by region and altitude (*p* = 0.0002 and *p* = 0.0005), and the total infection rates were affected by altitude (*p* = 0.018). However, the current infection rates of SFG *Rickettsia* showed no significant difference between yaks and Tibetan sheep, which implied that the infection of SFG *Rickettsia* might not be related to the host species in detected areas. So further research was needed to confirm this suspicion.

In conclusion, this study revealed the existence of SFG *Rickettsia* in tick vectors, yaks, and Tibetan sheep in QTPA, China. The phylogenetic analysis showed that all sequences obtained in the current study were closely related to the *R. raoultii* isolated from different tick species and domestic animals. Although the case of human infection by *R. raoultii* was not reported in the sampling regions, high infection rates should be paid more attention. Importantly, the relationship between SFG *Rickettsia*, tick species, and animal hosts should be further explored to understand their interrelationships.

## Data Availability Statement

The datasets presented in this study can be found in online repositories. The names of the repository/repositories and accession number(s) can be found in the article/supplementary material.

## Ethics Statement

The study was conducted in compliance with the ethical policies of the journal and the rules of the Ethics Committee of Qinghai University.

## Author Contributions

YL, Y-CH, J-XL, Y-LS, H-XH, J-SC, Y-HG, YW, PM, Y-PW, R-SL, W-KC, Z-HC, and JL designed the experiments and sampling. Y-CH, PM, Y-PW, R-SL, W-KC, and Z-HC performed the experiments. Y-CH and J-XL analyzed the data. Y-CH wrote the original manuscript. T-SQ, J-FY, Q-XZ, J-YC, Q-BZ, G-WH, MK, and YL reviewed the manuscript. All authors have read and agreed to the published version of the manuscript.

## Funding

This research was funded by the Regular Assistance Project of International Department of the Ministry of Science and Technology of China (Grant No. KY201904013), the Special Project for Scientific and Technological International Cooperation of the Science and Technology Department, Qinghai Province (2021-HZ-801), and the Veterinary Bureau Scientific Research Foundation of Qinghai Province (NMSY-2021-05).

## Conflict of Interest

The authors declare that the research was conducted in the absence of any commercial or financial relationships that could be construed as a potential conflict of interest.

## Publisher's Note

All claims expressed in this article are solely those of the authors and do not necessarily represent those of their affiliated organizations, or those of the publisher, the editors and the reviewers. Any product that may be evaluated in this article, or claim that may be made by its manufacturer, is not guaranteed or endorsed by the publisher.

## References

[1] Parola P Paddock CD Raoult D. Tick-borne rickettsioses around the world: emerging diseases challenging old concepts. Clin Microbiol Rev. (2005) 18:719–56. 10.1128/CMR.18.4.719-756.1 16223955 PMC1265907

[2] Merhej V Raoult D. Rickettsial evolution in the light of comparative genomics. Biol Rev Camb Philos Soc. (2011) 86:379–405. 10.1111/j.1469-185X.2010.00151.x 20716256

[3] Parola P Paddock CD Socolovschi C Labruna MB Mediannikov O Kernif T. Update on tick-borne rickettsioses around the world: a geographic approach. Clin Microbiol Rev. (2013) 26:657–702. 10.1128/CMR.00032-13 24092850 PMC3811236

[4] Piotrowski M Rymaszewska A. Expansion of Tick-Borne Rickettsioses in the World. Microorganisms. (2020) 8:1906. 10.3390/microorganisms8121906 33266186 PMC7760173

[5] Liu H Liang X Wang H Sun X Bai X Hu B. Molecular evidence of the spotted fever group Rickettsiae in ticks from Yunnan Province, Southwest China. Exp Appl Acarol. (2020) 80:339–48. 10.1007/s10493-020-00467-5 31925589

[6] Jian Y Li J Adjou Moumouni PF Zhang X Tumwebaze MA Wang G. Human spotted fever group *rickettsia* infecting yaks (*Bos grunniens*) in the Qinghai-Tibetan plateau area. Pathogens. (2020) 9:249. 10.3390/pathogens9040249 32231020 PMC7238049

[7] Han R Yang J Niu Q Liu Z Chen Z Kan W. Molecular prevalence of spotted fever group rickettsiae in ticks from Qinghai Province, northwestern China. Infect Genet Evol. (2018) 57:1–7. 10.1016/j.meegid.2017.10.025 29107656

[8] Shao JW Zhang XL Li WJ Huang HL Yan J. Distribution and molecular characterization of rickettsiae in ticks in Harbin area of Northeastern China. PLoS Negl Trop Dis. (2020) 14:e0008342. 10.1371/journal.pntd.0008342 32497120 PMC7272007

[9] Yang J Tian Z Liu Z Niu Q Han R Li Y. Novel spotted fever group rickettsiae in *Haemaphysalis qinghaiensis* ticks from Gansu, Northwest China. Parasit Vectors. (2016) 9:146. 10.1186/s13071-016-1423-7 26968160 PMC4788852

[10] Li K Shahzad M Zhang H Jiang X Mehmood K Zhao X. Socio-economic burden of parasitic infections in yaks from 1984 to 2017 on Qinghai Tibetan Plateau of China-A review. Acta Trop. (2018) 183:103–9. 10.1016/j.actatropica.2018.04.011 29626434

[11] Li J Jian Y Jia L Galon EM Benedicto B Wang G. Molecular characterization of tick-borne bacteria and protozoans in yaks (*Bos grunniens*), Tibetan sheep (*Ovis aries*) and Bactrian camels (*Camelus bactrianus*) in the Qinghai-Tibetan Plateau Area, China. Ticks Tick Borne Dis. (2020) 11:101466. 10.1016/j.ttbdis.2020.101466 32723655

[12] Gao J Luo J Fan R Fingerle V Guan G Liu Z. Cloning and characterization of a cDNA clone encoding calreticulin from *Haemaphysalis qinghaiensis* (Acari: Ixodidae). Parasitol Res. (2008) 102:737–46. 10.1007/s00436-007-0826-y 18087723

[13] Black WC. 4th, Piesman J. Phylogeny of hard- and soft-tick taxa (Acari: Ixodida) based on mitochondrial 16S rDNA sequences. Proc Natl Acad Sci U S A. (1994) 91:10034–8. 10.1073/pnas.91.21.10034 7937832 PMC44952

[14] Deng GF Jiang ZL. Economic insect fauna of China, Fasc 39 Acari: Ixodiate. Beijing: Science Press. (1991).

[15] Kidd L Maggi R Diniz PP Hegarty B Tucker M Breitschwerdt E. Evaluation of conventional and real-time PCR assays for detection and differentiation of Spotted Fever Group *Rickettsia* in dog blood. Vet Microbiol. (2008) 129:294–303. 10.1016/j.vetmic.2007.11.035 18226476

[16] Sekeyova Z Roux V Raoult D. Phylogeny of Rickettsia spp. inferred by comparing sequences of 'gene D', which encodes an intracytoplasmic protein. Int J Syst Evol Microbiol. (2001) 51:1353–60. 10.1099/00207713-51-4-1353 11491333

[17] Kumar S Stecher G. Tamura K. MEGA7: molecular evolutionary genetics analysis version 70 for bigger datasets. Mol Biol Evol. (2016) 33:1870–4. 10.1093/molbev/msw054 27004904 PMC8210823

[18] Chen Z Yang X Bu F Yang X Yang X Liu J. Ticks (acari: ixodoidea: argasidae, ixodidae) of China. Exp Appl Acarol. (2010) 51:393–404. 10.1007/s10493-010-9335-2 20101443

[19] Delord M Socolovschi C Parola P. Rickettsioses and Q fever in travelers (2004–2013). Travel Med Infect Dis. (2014) 12:443–58. 10.1016/j.tmaid.2014.08.006 25262433

[20] Socolovschi C Huynh TP Davoust B Gomez J Raoult D Parola P. Transovarial and trans-stadial transmission of Rickettsiae *africae* in *Amblyomma variegatum* ticks. Clin Microbiol Infect. (2009) 15:317–8. 10.1111/j.1469-0691.2008.02278.x 19456811

[21] Socolovschi C Matsumoto K Brouqui P Raoult D Parola P. Experimental infection of *Rhipicephalus sanguineus* with *Rickettsia conorii conorii*. Clin Microbiol Infect. (2009) 15:324–5. 10.1111/j.1469-0691.2008.02259.x 19438618

[22] Raoult D Roux V. Rickettsioses as paradigms of new or emerging infectious diseases. Clin Microbiol Rev. (1997) 10:694–719. 10.1128/CMR.10.4.694 9336669 PMC172941

[23] Paddock CD Sumner JW Comer JA Zaki SR Goldsmith CS Goddard J. *Rickettsia parkeri*: a newly recognized cause of spotted fever rickettsiosis in the United States. Clin Infect Dis. (2004) 38:805–11. 10.1086/381894 14999622

[24] Rydkina E Roux V Rudakov N Gafarova M Tarasevich I Raoult D. New Rickettsiae in ticks collected in territories of the Former Soviet Union. Emerg Infect Dis. (1999) 5:811–4. 10.3201/eid0506.990612 10603217 PMC2640811

[25] Mediannikov O Matsumoto K Samoylenko I Drancourt M Roux V Rydkina E. *Rickettsia raoultii* sp. nov, a spotted fever group rickettsia associated with Dermacentor ticks in Europe and Russia. Int J Syst Evol Microbiol. (2008) 58:1635–9. 10.1099/ijs.0.64952-0 18599708

[26] Tian ZC Liu GY Shen H Xie JR Luo J Tian MY. First report on the occurrence of *Rickettsia slovaca* and *Rickettsia raoultii* in *Dermacentor silvarum* in China. Parasit Vectors. (2012) 5:19. 10.1186/1756-3305-5-19 22257726 PMC3292836

[27] Wen J Jiao D Wang JH Yao DH Liu ZX Zhao G. *Rickettsia raoultii*, the predominant *Rickettsia* found in *Dermacentor silvarum* ticks in China-Russia border areas. Exp Appl Acarol. (2014) 63:579–85. 10.1007/s10493-014-9792-0 24699771

[28] Liu G Zhao S Tan W Hornok S Yuan W Mi L. Rickettsiae in red fox (*Vulpes vulpes*), marbled polecat (*Vormela peregusna*) and their ticks in northwestern China. Parasit Vectors. (2021) 14:204. 10.1186/s13071-021-04718-1 33874985 PMC8054388

[29] Parola P Rovery C Rolain JM Brouqui P Davoust B Raoult D. *Rickettsia slovaca* and *R*. raoultii in tick-borne Rickettsioses. Emerg Infect Dis. (2009) 15:1105–8. 10.3201/eid1507.081449 19624931 PMC2744242

[30] Jia N Zheng YC Ma L Huo QB Ni XB Jiang BG. Human infections with *Rickettsia raoultii*, China. Emerg Infect Dis. (2014) 20:866–8. 10.3201/eid2005.130995 24750663 PMC4012798

[31] Li H Zhang PH Huang Y Du J Cui N Yang ZD. Isolation and identification of *Rickettsia raoultii* in human cases: a surveillance study in 3 medical centers in China. Clin Infect Dis. (2018) 66:1109–15. 10.1093/cid/cix917 29069294

[32] Fournier PE Roux V Raoult D. Phylogenetic analysis of spotted fever group rickettsiae by study of the outer surface protein rOmpA. Int J Syst Bacteriol. (1998) 48:839–49. 10.1099/00207713-48-3-839 9734038

[33] Ishikura M Ando S Shinagawa Y Matsuura K Hasegawa S Nakayama T. Phylogenetic analysis of spotted fever group rickettsiae based on *gltA*, 17-kDa, and rOmpA genes amplified by nested PCR from ticks in Japan. Microbiol Immunol. (2003) 47:823–32. 10.1111/j.1348-0421.2003.tb03448.x 14638993

[34] Orkun Ö Karaer Z Çakmak A Nalbantoglu S. Spotted fever group rickettsiae in ticks in Turkey. Ticks Tick Borne Dis. (2014) 5:213–8. 10.1016/j.ttbdis.2012.11.018 24355764

[35] Taylor AJ Vongphayloth K Vongsouvath M Grandadam M Brey PT Newton PN. Large-Scale Survey for Tickborne Bacteria, Khammouan Province, Laos. Emerg Infect Dis. (2016) 22:1635–9. 10.3201/eid2209.151969 27532491 PMC4994350

[36] Jiang J Choi YJ Kim J Kim HC Klein TA Chong ST. Distribution of *Rickettsia* spp. in Ticks from Northwestern and Southwestern Provinces, Republic of Korea Korean. J Parasitol. (2019) 57:161–6. 10.3347/kjp.2019.57.2.161 31104408 PMC6526208

[37] Kooshki H Goudarzi G Faghihi F Telmadarraiy Z Edalat H Hosseini-Chegeni A. The first record of *Rickettsia hoogstraalii* (Rickettsiales: Rickettsiaceae) from *Argas persicus* (Acari: Argasidae) in Iran. Syst Appl Acarol. (2020) 25:1611–7. 10.11158/saa.25.9.7

[38] Nadim A Khanjani M Hosseini-Chegeni A Telmadarraiy Z. Identity and microbial agents related to *Dermacentor marginatus* Sulzer (Acari: Ixodidae) with a new record of *Rickettsia slovaca* (Rickettsiales: Rickettsiaceae) in Iran. Syst Appl Acarol. (2021) 26:367–78. 10.11158/saa.26.2.4

[39] Cao WC Zhan L De Vlas SJ Wen BH Yang H Richardus JH. Molecular detection of spotted fever group *Rickettsia* in *Dermacentor silvarum* from a forest area of northeastern China. J Med Entomol. (2008) 45:741–4. 10.1603/0022-2585(2008)45[741:mdosfg]2.0.co;2 18714877

[40] Guo WP Wang YH Lu Q Xu G Luo Y Ni X. Molecular detection of spotted fever group rickettsiae in hard ticks, northern China. Transbound Emerg Dis. (2019) 66:1587–96. 10.1111/tbed.13184 30920159

[41] Merhej V Angelakis E Socolovschi C Raoult D. Genotyping, evolution and epidemiological findings of *Rickettsia* species. Infect Genet Evol. (2014) 25:122–37. 10.1016/j.meegid.2014.03.014 24662440

[42] Ando S Kurosawa M Sakata A Fujita H Sakai K Sekine M. Human *Rickettsia heilongjiangensis* infection, Japan. Emerg Infect Dis. (2010) 16:1306–8. 10.3201/eid1608.100049 20678332 PMC3298298

[43] Chung MH Lee SH Kim MJ Lee JH Kim ES Lee JS. Japanese spotted fever, South Korea. Emerg Infect Dis. (2006) 12:1122–4. 10.3201/eid1207.051372 16836831 PMC3291047

[44] Takada N Fujita H Kawabata H Ando S Sakata A Takano A. Spotted fever group *Rickettsia* sp. closely related to Rickettsia japonica, Thailand. Emerg Infect Dis. (2009) 15:610–1. 10.3201/eid1504.071271 19331747 PMC2671449

[45] Keskin A Bursali A Keskin A Tekin S. Molecular detection of spotted fever group rickettsiae in ticks removed from humans in Turkey. Ticks Tick Borne Dis. (2016) 7:951–3. 10.1016/j.ttbdis.2016.04.015 27131413

[46] Hosseini-Chegeni A Tavakoli M Telmadarraiy Z. The updated list of ticks (Acari: Ixodidae & Argasidae) occurring in Iran with a key to the identification of species. Syst Appl Acarol. (2019) 24:2133–66. 10.11158/saa.24.11.8

[47] Hosseini-Chegeni A Tavakoli M Telmadarraiy Z Faghihi F. Molecular detection of spotted fever group *Rickettsia* (Rickettsiales: Rickettsiaceae) in ticks of Iran. Arch Razi Inst. (2020) 75:317–25. 10.22092/ari.2019.125746.1317 33025772 PMC8418809

[48] Samoylenko I Shpynov S Raoult D Rudakov N Fournier PE. Evaluation of Dermacentor species naturally infected with Rickettsia raoultii. Clin Microbiol Infect. (2009) 15:305–6. 10.1111/j.1469-0691.2008.02249.x 19438650

